# Accuracy of STOP-Bang Questionnaire in Predicting Difficult Mask Ventilation: An Observational Study

**DOI:** 10.7759/cureus.15955

**Published:** 2021-06-27

**Authors:** Marium N Khan, Aliya Ahmed

**Affiliations:** 1 Anaesthetics, Blackpool Victoria Teaching Hospital, Blackpool, GBR; 2 Anaesthesiology, Aga Khan University, Karachi, PAK

**Keywords:** difficult mask ventilation, stop-bang score, difficult airway, general anesthesia, preoperative assessment

## Abstract

Introduction

Difficulty with bag-mask ventilation after the induction of general anesthesia and muscle relaxation places the patient at risk for a prolonged period of apnea and hypoxia and thus, at an increased risk of morbidity and mortality. This study was designed to assess the accuracy of the STOP-Bang questionnaire in predicting difficult mask ventilation (DMV) in patients receiving general anesthesia for elective surgical procedures.

Methods

It was a prospective cross-sectional, observational study conducted at a university teaching hospital. A total of 530 patients undergoing surgery under general anesthesia with endotracheal intubation were enrolled. STOP-Bang questionnaire was filled at pre-operative anesthesia assessment. Ease or difficulty of mask ventilation was assessed and documented by a senior resident responsible for intraoperative anesthetic management.

Results

Out of 530 patients, 139 (26.22%) had a STOP-Bang score of ≥ 3, of whom 55 (39.5%) were found to have DMV. Out of 391 patients with a STOP-Bang score of < 3, only 29 patients (7.5%) had DMV (P ≤0.001). Snoring, high blood pressure, BMI more than 35 kg/m^2^, age more than 50 years, neck circumference more than 40 cm, and male gender were significantly associated with DMV. The accuracy of the STOP-Bang questionnaire in predicting difficult mask ventilation was 78.68% (95% CI 74.99-81.95) with a negative predictive value of 92.58%. The sensitivity and specificity were found to be 65.48% and 81.17% respectively.

Conclusion

STOP-Bang score has a high negative predictive value and can be very useful in ruling out the possibility of difficult mask ventilation.

## Introduction

Optimal oxygenation and ventilation of an anesthetized patient is the core responsibility of anesthesiologists and any difficulty in airway management is a major cause for concern. At the induction of anesthesia, neuromuscular blocking agents are commonly administered, which leads to muscle relaxation and apnea. The anesthesiologist performs mask ventilation for 3-4 minutes until the muscle relaxant takes its effect. Endotracheal intubation is then accomplished. Effective mask ventilation is therefore crucial for the initial 3-4 minutes before endotracheal intubation. Difficulties in mask ventilation place the patient at risk of prolonged apnea, with resulting hypoxia.

There are several factors that can predict possible difficulty in mask ventilation, obstructive sleep apnea (OSA) being one such factor [1]. Research has suggested that a high risk for OSA might be a predictor of difficulty in airway management during anesthesia [2-4]. However, patients suffering from OSA might be undiagnosed when they present for surgery [1,5]. The STOP-Bang questionnaire is a screening tool for assessing the risk for OSA [6,7]. Since it has been shown that OSA patients have a higher incidence of difficulty in mask ventilation [2,8], it seems logical to assume that a high STOP-Bang score might be useful as an independent predictor of difficult mask ventilation.

A study on obese patients has shown that the STOP-Bang questionnaire is useful in predicting difficult intubation in these patients [8]. Identifying patients at an increased risk of OSA by assessing the STOP-Bang score during pre-anesthesia assessment and being prepared accordingly for possible difficulties in airway management with relevant armamentarium at hand would be useful in preventing adverse events related to difficult mask ventilation (DMV) and difficult endotracheal intubation [9]. Despite the globally growing recognition of the association of OSA with difficult airway management, available data about the relationship of high STOP-Bang score with DMV is scarce. In this study, we hypothesized that a high STOP-Bang score will be useful in predicting DMV and assessed the accuracy of the STOP-Bang questionnaire in predicting DMV in adult surgical patients.

## Materials and methods

Approval for the study was granted by the Ethics Review Committee of Aga Khan University (approval number: 4313-Ane-ERC-16). Patients included in the study comprised of those aged 18-60 years, having American Society of Anesthesiologists (ASA) status I-III, scheduled for elective surgery under general anesthesia and endotracheal intubation. Patients who refused to participate, those having neuromuscular disease, facial abnormalities and patients undergoing cardiothoracic, head and neck, obstetric, pediatric, neurosurgery, emergency surgery, or patients previously diagnosed with OSA (by polysomnography), having any airway related anatomical deformity (e.g. Down’s syndrome) or pathological condition such as a history of head/neck surgery or radiation were excluded. Written informed consent was acquired from all study participants.

Eight easily administrable questions are included in the STOP-Bang questionnaire, each having a yes/no response (the questions are regarding snoring, tiredness or sleepiness during daytime, breath-holding during sleep, high blood pressure, body mass index (BMI), age, neck circumference and gender). A ‘yes’ response is given one mark and a ‘no’ represents zero. The marks are added up to obtain the final STOP-Bang score. The possible range of score is zero to eight.

The study participants were recruited from the scheduled elective surgical lists. The demographic data and STOP-Bang scores for each study participant who satisfied the inclusion criteria were recorded at the time of pre-anesthesia assessment by the resident assessing the patient. A score of ≥ 3 was considered as an increased risk for OSA and < 3 as having a low risk. Intra-operative observations were recorded in the study form by the anesthesia resident anesthetizing the patient under the supervision of a consultant anesthesiologist. It was ensured that the resident performing bag-mask ventilation on the study subjects had more than two years of anesthesia experience. The questionnaire and intra-operative study form were collected by the primary investigator from the residents. The anesthesiologist responsible for airway management was blinded to the STOP-Bang score.

Standard monitoring (electrocardiogram, non-invasive blood pressure, and peripheral oxygen saturation) was applied and baseline readings were noted. Pre-oxygenation was done for three minutes and induction of anesthesia was conducted with morphine 0.1 mg/kg and propofol 1-2 mg/kg until the patient became unresponsive to verbal communication, followed by the administration of atracurium 0.5mg/kg. Mask ventilation was then performed for three minutes using the circle system with the patient’s head in a standard sniffing position. Mask ventilation was defined as easy when visible chest rise was achieved with the appearance of end-tidal carbon dioxide graph by a single anesthesiologist, and difficult if there was no chest rise and absent capnograph or peripheral oxygen saturation dropped to 90% or less despite the use of airway adjuncts (oral or nasal pharyngeal airway) or when there was the need for two providers to achieve mask ventilation [9]. If the resident could not mask ventilate the patient, it was the supervising consultant’s decision when to intervene and take over. After three minutes of mask ventilation, the trachea was intubated using a Macintosh laryngoscope. The correct position of the endotracheal tube was confirmed by monitoring of end-tidal carbon dioxide and bilateral auscultation of the chest.

IBM Corp. Released 2010. IBM SPSS Statistics for Windows, Version 19.0. Armonk, NY: IBM Corp. was used for data analysis. Mean and standard deviation was used to express quantitative variables such as age, BMI, and STOP-Bang score. Qualitative observations such as gender, ASA status, STOP-Bang score (≥3 & < 3), and difficult mask ventilation were presented as numbers and percentages. Chi-square test was used to observe the association between factors assessed and DMV and crude odds ratios were computed for each factor. Multivariate logistic stepwise regression analysis was employed to compute the adjusted odds ratio and significant contribution of the predictors. P-value ≤0.05 was taken as significant. Accuracy was computed for STOP-Bang Score in predicting difficult mask ventilation. The area under the receiver operating characteristic curve (AUC-ROC) was used to determine the clinical value of the score. A value of 0.5 under the receiver operating characteristic (ROC) curve demonstrated that the performance of the variable was no better than chance, while a reading of 1.0 signified that the discrimination was perfect.

Sample size calculation was performed using MedCalc for Windows version 18.5 (MedCalc Software Ltd., Ostend, Belgium), taking into account the study performed by Corso et al., which reported an incidence of DMV of 23% with three or more risk factors signifying a high risk of OSA [2]. Five hundred and twelve patients were required to detect an improvement of discriminating power by the area under the curve (AUC) when 15% absolute difference (e.g., from 50% to 65%) was considered clinically significant with 90% power and 1% type I error. Finally, a total of 530 patients were recruited for this study.

## Results

This prospective, observational study included 530 patients. The mean age and BMI of the patients was 39.89 ±13.99 years and 27.135 ± 5.54 kg/m2 respectively. There were 302 (57%) female and 228 (43%) male patients. Regarding ASA status, 248 (46.8%) belonged to ASA-I, 245 (46.2%) to ASA-II and 37 (7%) to ASA-III status.

Overall, DMV was found in 84 patients (15.8%). Reasons/factors associated with DMV are provided in Table 1. Out of the 530 patients, 139 (26.22%) had a STOP-Bang score of ≥ 3, while 391 (73.8%) had a score of < 3. Among the 139 patients with a score of ≥ 3, 55 (39.5%) were found to have DMV. Whereas, out of the 391 patients with a score of < 3, only 29 patients (7.5%) had DMV. This difference was statistically significant (P ≤0.001). Unadjusted odds ratio showed that all factors of the STOP-Bang criteria had two to eight times more likely association with DMV, while on multivariate analysis snoring, high blood pressure, BMI more than 35 kg/m2, age more than 50 years, neck circumference more than 40 cm and male gender were significantly associated with DMV as presented in Table 1.

**Table 1:** Univariate and multivariable analysis showing association between STOP-Bang criteria and difficult mask ventilation (n=530) DMV: difficult mask ventilation; UOR: unadjusted odds ratio; aOR: adjusted odds ratio; CI: confidence interval

Variables	N	DMV n=84 (15.8%)	P-Value	UOR (95% CI)	P-Value	aOR (95% CI)
Snoring						
Yes	173	58 (33.5%)	0.0005	6.42 (3.86-10.68)	0.0005	5.32 (2.93-9.64)
No	357	26 (7.3%)				
Tired during daytime						
Yes	96	25 (26%)	0.0005	2.24 (1.31-3.81)	0.687	1.16 (0.56-2.39)
No	434	59 (13.6%)				
Observed apnoea						
Yes	31	12 (38.7%)	0.0005	3.74 (1.74-8.05)	0.550	1.40 (0.46-4.25)
No	499	72 (14.4%)				
High blood pressure						
Yes	167	47 (28.1%)	0.0005	3.45 (2.14-5.57)	0.007	2.32 (1.26-4.27)
No	363	37 (10.2%)				
BMI >35 kg						
Yes	45	24 (53.3%)	0.0005	8.09 (4.25-15.43)	0.0005	4.62 (2.11-10.08)
No	485	60 (12.4%)				
Age >50 years						
Yes	125	42 (33.6%)	0.0005	4.37 (2.68-7.14)	0.0005	3.25 (1.75-9.02)
No	405	42 (10.4%)				
Neck circumference > 40 cm						
Yes	43	19 (44.2%)	0.0005	5.14 (2.67-9.91)	0.003	3.42 (1.45-7.82)
No	487	65 (13.3%)				
Gender Male						
Yes	218	46 (21.1%)	0.0005	1.93 (1.21-3.08)	0.021	2.03 (1.11-3.72)
No	312	38 (12.2%)				

**Figure 1:**
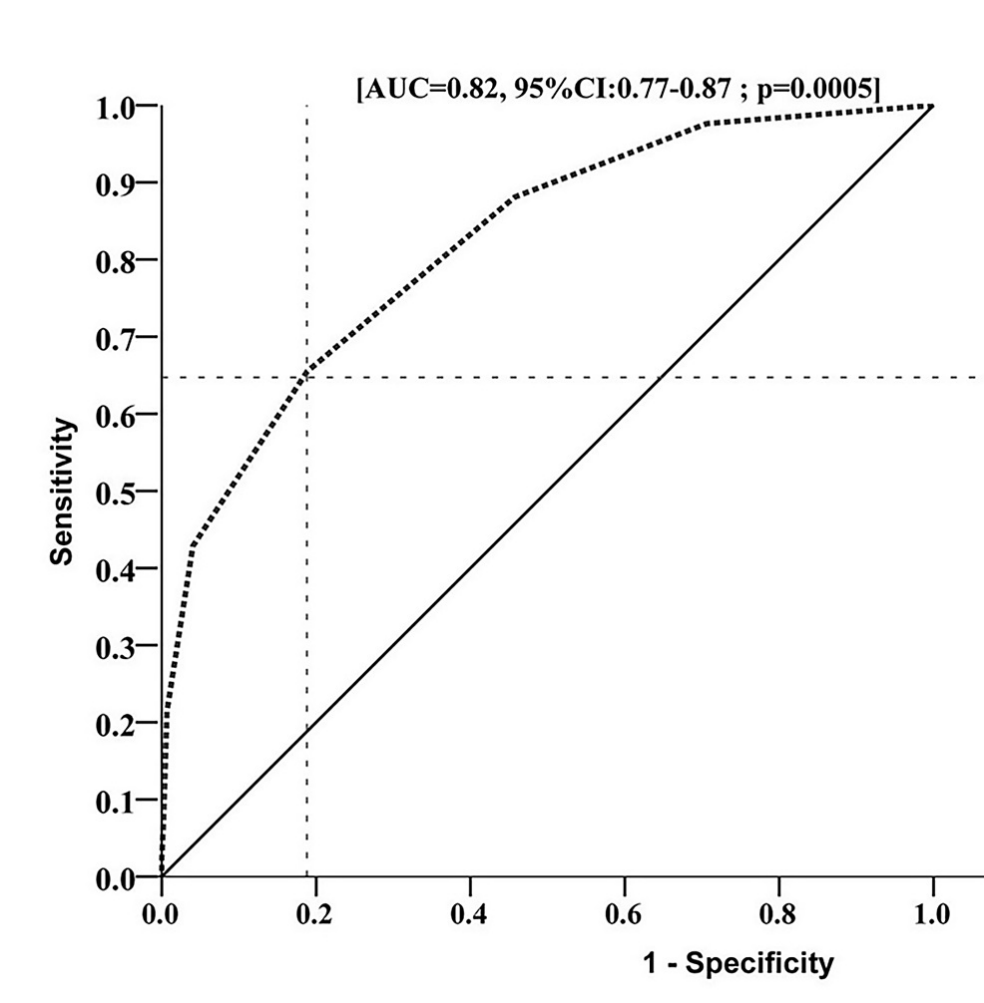
Receiver operating characteristic (ROC) curve showing ability of STOP-Bang score in predicting difficult mask ventilation AUC: Area under the curve

According to our results, the accuracy of STOP-Bang questionnaire in predicting difficult mask ventilation was 78.68% (95% CI: 74.99-81.95), with a negative predictive value of 92.58% (95% CI: 89.55-94.79). The sensitivity and specificity of the questionnaire in predicting DMV was found to be 65.48% and 81.17%, respectively (Table 2). ROC plot in Figure 1 shows the AUC which is 0.82 (95% CI: 0.77-0.87). The maximum sensitivity and minimum specificity was observed at the optimal cut-off point of STOP-Bang score of ≥3.

**Table 2:** Accuracy of STOP-Bang scores in predicting difficult mask ventilation True Positive: 55; False Positive: 84; False Negative: 29; True Negative: 362; Cutoff: Stop Bang ≥ 3; Prevalence of DMV: 15.8% (84/530)

Parameter	Stop Bang ≥ 3	
	Estimates	(95% CI)
Sensitivity	65.48%	(54.83-74.77)
Specificity	81.17%	(77.28-84.52)
Positive Predictive Value	39.57%	(31.82- 47.87)
Negative Predictive Value	92.58%	(89.55- 94.79)
Diagnostic Accuracy	78.68%	(74.99- 81.95)
Cohen's kappa	0.37	(0.28 - 0.45)

## Discussion

Mask ventilation is an essential skill that needs to be mastered by all anesthesiologists. It is an integral part of the majority of general anesthesia inductions and is a crucial technique to resort to for maintaining oxygenation during a failed or difficult intubation. In our prospective observational study on 530 patients, an overall frequency of DMV was found to be 15.8%. Because of a lack of consensus on its definition, the reported incidence of DMV shows a wide disparity, ranging from 0.08% to 15% [3,4,10,11]. Our result of 15.8% corresponds to the higher end of this range. A reason for this disparity might be the possible racial anatomical variations that can lead to DMV and difficult intubation in some populations [12]. Difficult or impossible mask ventilation has been found to be an important cause of permanent brain damage or even death during anesthesia [13]. Furthermore, it has been assessed that adult patients with DMV have a fourfold increased risk of difficult intubation [14]. Careful airway assessment and prediction of possible DMV are essential in devising a successful airway management plan to prevent adverse events and enhance patient safety. Thus, the anticipation of difficult airway and appropriate preparation can potentially decrease the serious consequences of hypoxia associated with failure of effective ventilation.

Our study showed that 39.5% of patients with a STOP-Bang score of ≥ 3 had DMV compared to 7.5% in patients with a score of < 3. In their study on 1399 patients, Cattano et al. endeavored to identify the predictive factors for DMV by conducting a retrospective subgroup analysis of patients’ preoperative airway assessment before elective surgery [10]. According to their results, those with a history of OSA had a 17% incidence of DMV. Our patients, in comparison, were not diagnosed OSA patients and our assessment was based on their STOP-Bang scores determined at preoperative assessment, which was not available in their retrospective analysis. The STOP-Bang questionnaire is a validated, easy-to-use tool to screen for OSA during the peri-operative period [15,16]. The scores obtained are used to identify the risk for OSA, presence of three or more criteria being considered an increased risk for OSA. Patients with OSA have episodes of upper airway obstruction during sleep with repeated arousals and desaturations [17]. Peri-operative respiratory complications are one of the most significant features of OSA that concerns the anesthesiologist and it has been identified as an independent predictor of difficult intubation [18]. DMV in patients with OSA may be explained by various factors, including tonsillar hyperplasia, a large tongue, pharyngeal wall collapse caused by redundant tissue, and increased neck circumference [11].

Out of the eight criteria of the STOP-Bang questionnaire, we found six criteria to be independently associated with a risk of DMV (P = 0.0005), including snoring, high blood pressure, BMI >35 kg/m2, age > 50 years, neck circumference > 40 cm and male gender. The criteria identified by Cattano et al. as risk factors for DMV included age ≥ 47 years, history of difficult intubation, BMI ≥ 35 kg/m2, perceived short neck, neck circumference ≥ 40 cm, presence of facial hair, and history of OSA [10]. Langerton et al., in their prospective study on 1502 patients identified five independent criteria for DMV including age > 55 years, BMI > 26 kg/m2, a beard, absence of teeth, and history of snoring [19]. As we assessed only the STOP-Bang criteria, the presence of facial hair and the absence of teeth were not included in our data analysis.

It has been seen that approximately 24% of surgical patients are affected with OSA [15,20]. A large majority of patients having OSA remain undiagnosed [9]. For this reason, preoperative screening for OSA has been recommended by the Society of Anesthesia and Sleep Medicine and the American Society of Anesthesiologists [20,21]. High sensitivity for detection of OSA has been shown with a STOP-Bang score of ≥3 [21]. However, the specificity is lower, resulting in a high false-negative rate. In their prospective observational trial including 22,660 anesthetics, Khetarpal et al. have reported that OSA is an independent predictor of difficult and impossible mask ventilation [3]. In our study, 139 (26.2%) patients had a STOP-Bang score ≥ 3. Out of these 139 patients, 55, (39.5%) exhibited difficulty in mask ventilation. In contrast, Corso et al. have demonstrated an incidence of DMV of 23% in high OSA risk patients, but they used ≥5 as the cut-off score to avoid false-negative results [2]. We used a score of ≥ 3 for high risk of OSA as it has shown a high sensitivity and is recommended by the developers of the questionnaire [21]. Toshniwal et al. have reported that morbidly obese patients having a STOP-Bang score of ≥ 3 demonstrated an increased risk for difficulty in airway management [8]. They also reported that muscle relaxation led to an improvement in mask ventilation in 43.9% of obese patients with DMV and 17% of these patients turned out to have difficult intubation as well. However, a STOP-Bang score of < 3 does not completely rule out the possibility of encountering difficult mask ventilation. Although 391/530 patients in our study were low-risk for OSA (STOP-Bang < 3), 29 (7.42%) of these patients had difficult mask ventilation.

In our study, the accuracy of the STOP-Bang questionnaire in predicting difficult mask ventilation was found to be 78.68% (95% CI: 74.99-81.95), with a negative predictive value of 92.58% (95% CI: 89.55-94.79). Although no direct comparison is available, our data show moderately high accuracy of STOP-Bang score for predicting DMV, while the negative predictive value is even higher. For a cut-off score of ≥ 3, the sensitivity and specificity of the STOP-Bang questionnaire in predicting DMV was 65.48% and 81.17%, respectively and the area under the ROC curve was 0.82. This indicates that preoperative scoring of STOP-Bang criteria would be useful in predicting difficult mask ventilation and thus alerting the anesthesiologist towards taking appropriate safety measures. Although a lot of work has been done to identify criteria for predicting difficult intubation and practice guidelines and algorithms have been developed to reduce the incidence of potential adverse outcomes, similar work has not been done to identify factors that can predict DMV [19]. A very dangerous situation can develop if mask ventilation is inadequate in a patient in whom intubation turns out to be difficult or impossible. Therefore it is essential for patient safety to identify criteria that can alert the anesthesiologist towards the possibility of DMV.

OSA prevalence varies with the type of surgery, being highest among bariatric surgery patients [22]. Singh et al. in a cohort study on 819 patients demonstrated that anesthesiologists and surgeons neglected to identify many patients with pre-existing OSA prior to surgery [23]. It is crucial for anesthesiologists to assess patients undergoing surgery for OSA in order to devise an appropriate plan for the peri-operative care of such patients, owing to the mortality associated with it. Hiremath et al. demonstrated a strong association between diagnosed OSA and difficult intubation [24]. Plunkett et al have reported that, in their prospective study, nine out of ten patients with difficult mask ventilation at induction were proven to be suffering from OSA [25]. Due to differences in study designs, it is hard to compare our findings with those of the other authors. Some other studies also report that difficult mask ventilation was encountered more frequently in high-risk OSA patients, but either the STOP-Bang cut-off score used (≥5) or the patient cohort (morbidly obese patients, diagnosed OSA patients, etc.) differed from our study population [2,7]. The hypothesis of our study was that the STOP-Bang questionnaire is useful in predicting DMV; however, our results demonstrate its high negative predictive value in ruling out DMV among surgical patients.

A limitation of this study is that other difficult airway predictors, such as Mallampati score, inter-incisor distance, and jaw movements were not analyzed, although they are evaluated in all surgical patients during a routine pre-anesthetic assessment at the authors’ center. Evaluating these criteria, along with the STOP-Bang score during pre-anesthetic assessment would further enhance the value of STOP-Bang scores in the prediction of difficulty in airway management.

## Conclusions

Our study has demonstrated that the STOP-Bang questionnaire has a high (92%) negative predictive value for DMV. It can therefore be very helpful in ruling out the possibility of DMV. The questionnaire has been found to be 78% accurate in predicting DMV at the cut-off score of ≥3. AUC was observed to be high due to the high rate of true negative values. We recommend the conduct of future studies with a higher cut-off STOP-Bang score and to combine it with other predictors of the difficult airway to assess its value in predicting DMV and to further consolidate our findings of its high negative predictive value.
